# Treatment Strategies in Diffuse Midline Gliomas With the H3K27M Mutation: The Role of Convection-Enhanced Delivery in Overcoming Anatomic Challenges

**DOI:** 10.3389/fonc.2019.00031

**Published:** 2019-02-08

**Authors:** Benjamin T. Himes, Liang Zhang, David J. Daniels

**Affiliations:** ^1^Department of Neurosurgery, Mayo Clinic, Rochester, MN, United States; ^2^Department of Molecular Pharmacology and Experimental Therapeutics, Mayo Clinic, Rochester, MN, United States

**Keywords:** diffuse intrinsic pontine glioma (DIPG), convection-enhanced delivery (CED), H3K27M mutation, blood brain barrier (BBB), alternative delivery method

## Abstract

Diffuse midline gliomas harboring the H3 K27M mutation—including the previously named diffuse intrinsic pontine glioma (DIPG)—are lethal high-grade pediatric brain tumors that are inoperable and without cure. Despite numerous clinical trials, the prognosis remains poor, with a median survival of ~1 year from diagnosis. Systemic administration of chemotherapeutic agents is often hindered by the blood brain barrier (BBB), and even drugs that successfully cross the barrier may suffer from unpredictable distributions. The challenge in treating this deadly disease relies on effective delivery of a therapeutic agent to the bulk tumor as well as infiltrating cells. Therefore, methods that can enhance drug delivery to the brain are of great interest. Convection-enhanced delivery (CED) is a strategy that bypasses the BBB entirely and enhances drug distribution by applying hydraulic pressure to deliver agents directly and evenly into a target region. This technique reliably distributes infusate homogenously through the interstitial space of the target region and achieves high local drug concentrations in the brain. Moreover, recent studies have also shown that continuous delivery of drug over an extended period of time is safe, feasible, and more efficacious than standard single session CED. Therefore, CED represents a promising technique for treating midline tumors with the H3K27M mutation.

## Introduction

### Clinical Presentation of Diffuse Intrinsic Pontine Glioma (DIPG)

**Figure 1:**
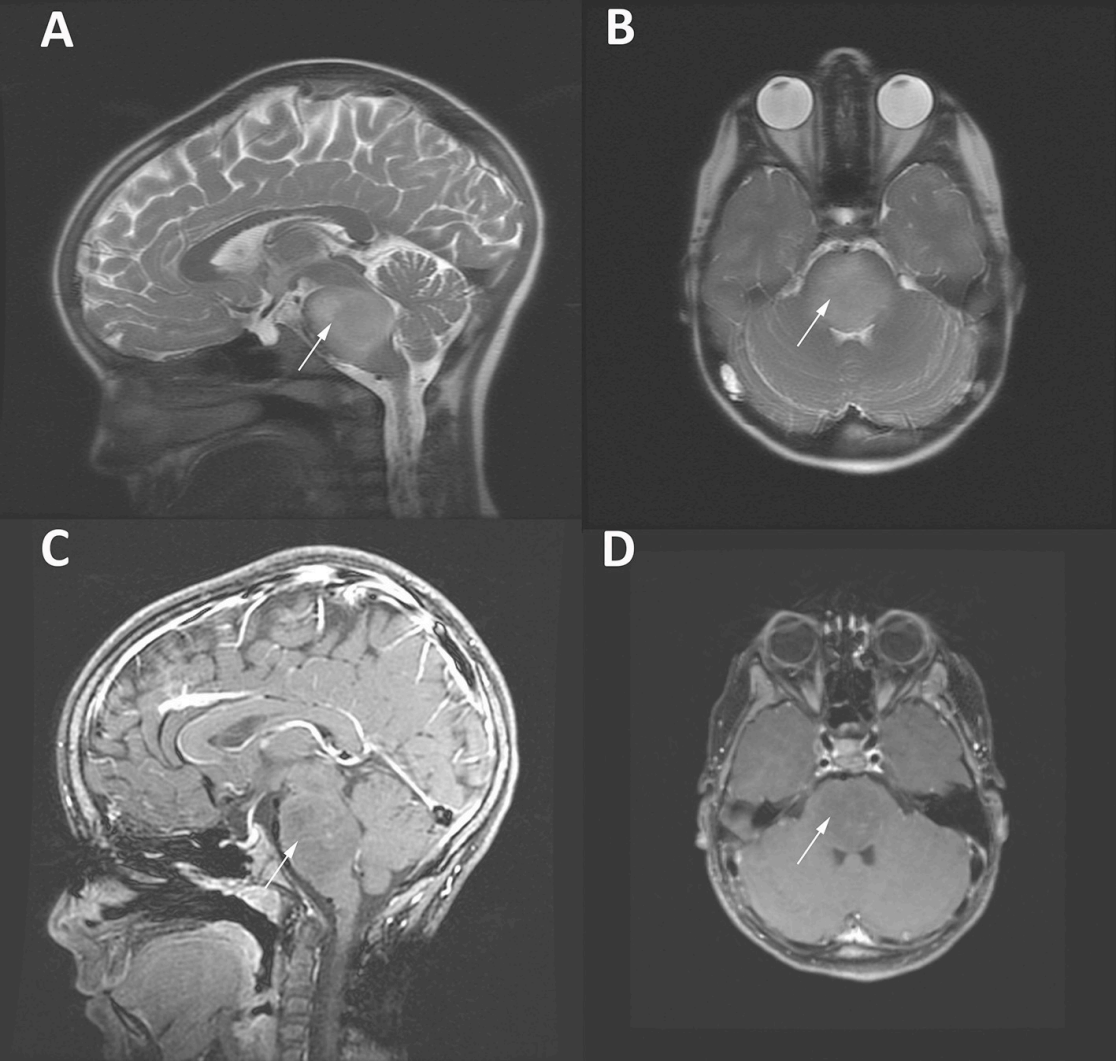
MRI imaging of an 8 year-old girl with a DIPG tumor (white arrows). T2-weighted sagittal **(A)** and axial **(B)** images demonstrate the enlargement of the brainstem and highlight the diffuse infiltrative characteristic of DIPG tumors. **(C,D)** Gadolinium-enhanced T1-weighted MRI images of the same patient demonstrating scant patchy enhancement.

Primary pediatric brain tumors are rare entities, with an incidence of ~2,200 cases annually (1–3). Diffuse intrinsic pontine glioma (DIPG), which makes up ~20% of these primary pediatric primary brain tumors, carries among the direst prognosis (4, 5). A diffusely infiltrative lesion situated in the brainstem of children, these tumors often present with a constellation of symptoms including headache, nausea, cranial nerve dysfunction, cerebellar signs, and long tract sings, with some patients demonstrating hydrocephalus (6, 7). These tumors occur primarily at a median age of seven (8). DIPGs are one of the few central nervous system (CNS) neoplasms for which diagnosis can be made with radiographic imaging alone, as the diffuse, non-enhancing T2 signal change in the brainstem, encompassing over half of the pons, is so highly characteristic and biopsy of the lesion carries risk of neurologic deficit (Figure 1) (6). However, recent reports have shown that biopsies of these tumors are safe, and molecular analysis from this tissue has greatly increased our understanding of the unique tumor biology (9, 10). Prognosis of these tumors remains uniformly poor, with a median survival of around 1 year from the time of diagnosis despite extensive efforts to improve this (4, 11, 12). Patients eventually develop worsening neurologic deficits, brainstem dysfunction, and hydrocephalus, before ultimately succumbing to their disease.

### Current Therapies

The anatomic location of the tumor severely limits any opportunity for meaningful surgical resection, and treatment usually consists of standard fractionated radiation to a dose of 54-59 Gy (over 30 fractions) (6). Multiple regimens involving monotherapy and combination chemotherapy have been trialed, with uniformly poor results (1, 4, 13). More recently, advances have been made in the field of chimeric-antigen receptor (CAR) T cells as a targeted therapy targeting anti-GD2 for DIPG; however, these studies remain in early stages (14). With these limited treatment options, there remains a critical need to develop novel therapeutics and effective delivery mechanisms for DIPG.

### H3K27M Mutation

Several key mutations appear to define these tumors. The substitution of a lysine for methionine at position 27 in histone H3 (specifically in either histone 3.3 or 3.1 genes) resulting in a H3K27M mutation, is the most commonly found mutation, and is associated with a worse prognosis than wild-type tumors (15–17). In the largest study of classically defined DIPG tumors that have been biopsied in the molecular era (91 patients), researchers in France found all but one tumor had either a somatic mutation in H3K27M and/or loss of H3K27 trimethylation, highlighting the importance of histone H3 in the pathology of this disease (10). This has implications for chromatin remodeling on a wide scale, with epigenetic silencing and activation of various elements of the genome broadly impacted, as the lysine 27 residue is a critical site for epigenetic regulation (18, 19). This mutation is sufficiently characteristic of these tumors that the World Health Organization recently redefined these tumors as “diffuse midline glioma, H3K27M mutant” in the latest criteria (6, 20). For simplicity and historical reasons we will continue to use the term DIPG tumors throughout this review. Crucially, this single histone mutation and subsequent epigenetic changes presents a potentially druggable target for the treatment of DIPG, and its prevalent expression in DIPG implies an essential role in tumorigenesis and growth, further raising its appeal as a therapeutic target (10, 16). Drugs that modify the histone epigenome have recently been identified as promising targeted therapies including the Histone Deacetylase (HDAC) inhibitor panobinostat and the bromodomain inhibitor (JQ1) have shown some early evidence as promising targeted therapies (21–23). However, the anatomic location of these tumors presents a challenge for effective delivery of medications, requiring novel drug delivery strategies.

## The Blood-Brain Barrier (BBB)

Drug delivery to lesions in the brain presents a number of specific challenges; chief among them is getting drugs past the blood brain barrier (BBB). The BBB, a unique tissue-specific modification of the capillary endothelium and basal lamina, serves to exclude nearly all macro molecules and most small molecules from extravagating into the brain parenchyma (24). Highly polar or charged particles are excluded, as are molecules as small as 100 Da (24). This poses obvious challenges in systemic administration of drug, requiring that molecules or mechanisms of drug delivery be specifically engineered in order to bypass the BBB. Even in the case of tumors with significant contrast enhancement (such as glioblastoma), indicative of BBB disruption, effective delivery of drug through the systemic circulation remains a challenge (25). The BBB in cases of DIPG is frequently preserved, as evidenced by the general lack of enhancement in these tumors (26). Further, the BBB is variable throughout the CNS, with some areas (such as the circumventricular organs) that have a reduced or absent barrier (27). In contrast, there is some evidence that the brainstem may be home to an even more robust BBB, further restricting the range of drugs that may be effectively delivered to the region. Using dynamic contrast-enhanced MRI, Subashi et al. demonstrated reduced BBB permeability in brainstem gliomas relative to identical tumors implanted supratentorially in a mouse model (28). There is also some evidence, particularly in mice, that the brainstem has a lower density of capillaries than cortical regions or basal ganglia, which would also imply increased difficulty in delivering effective therapeutic payloads through the circulation (29, 30).

## Alternative Delivery Methods

Given the challenges presented by the BBB, significant effort has been put into finding means to bypass or disrupt the BBB in a controlled fashion for enhanced delivery of therapeutics. Direct intracranial delivery provides an attractive means to circumvent the BBB, as surgical resection remains the mainstay of treatment of many brain tumors and presents an opportunity for direct inoculation of therapeutic agents into the brain parenchyma. Carmustine wafers have been one such technology, though their efficacy and degree of tissue penetration are somewhat limited (31–34). Such an approach is of limited use in tumors with limited surgical accessibility, including DIPG. Intra-arterial (IA) infusion of therapeutics is an area of active research, as such a route of administration circumvents fist-pass metabolism by the liver, broadening the scope of pharmacologic tools available to cross the BBB. IA therapy also allows for selective infusion of medication into end-arteries in the brain, allowing for administration of higher dosages of chemotherapy than can be administered systemically (35, 36). Certain transport proteins important for BBB function, including P-gp, are also expressed at lower levels on the arteriolar side of the circulation (29). Co-administration of mannitol with IA chemotherapy has been investigated as a means of crossing the BBB (36, 37). Often studied in conjunction with an IA delivery mechanism, focused ultrasound has shown some promise in circumventing the BBB (38–40). However, this technique has proven inconsistent (41). Infusion of microbubbles coupled with focused ultrasound can allow for focused disruption of the BBB, allowing medications to temporarily cross (42). Alli et al. recently demonstrated the feasibility of this technique in disrupting the BBB to allow for increased local delivery of doxorubicin (43). Some drugs may also be loaded into these microbubbles, creating a packaging system the protect drugs until they reach their destination, providing a mechanism to control their release in a specified location (44). Intranasal delivery has also been advanced as means of improving drug delivery to the brain, though such a route precludes targeting toward specific brain regions (45).

## Convection-Enhanced Delivery

Convection-enhanced delivery (CED) is a therapeutic strategy that addresses some of the key pitfalls in the treatment of brain tumors. It allows for targeted treatment of a specific region via a cannula that can be placed in difficult to access areas, and allows for direct intraparenchymal infusion of drug, bypassing the BBB. Fundamentally, CED is the process of continuously infusion drug at a steady rate over a prolonged period of time, allowing a constant pressure head to drive infusate penetration into surrounding tissue via bulk flow and avoid reflux into the infusing cannula, treating a spherical or elliptical region of tissue (46). In this way a small point of access can be used to treat a relatively large volume of tissue, an appealing characteristic for treating tumors in privileged locations such as DIPG. Further, infusion via CED proceeds in a highly predictable fashion, with a sharp drop-off in drug dosage beyond the predicted volume of the infusate, makes it ideal for treating a specific region while avoiding treatment of uninvolved surrounding structures (46, 47). Infusion in this manner proceeds best along white matter tracts, which would likely be of benefit in treating DIPG (48, 49).

### Catheter Placement

**Figure 2:**
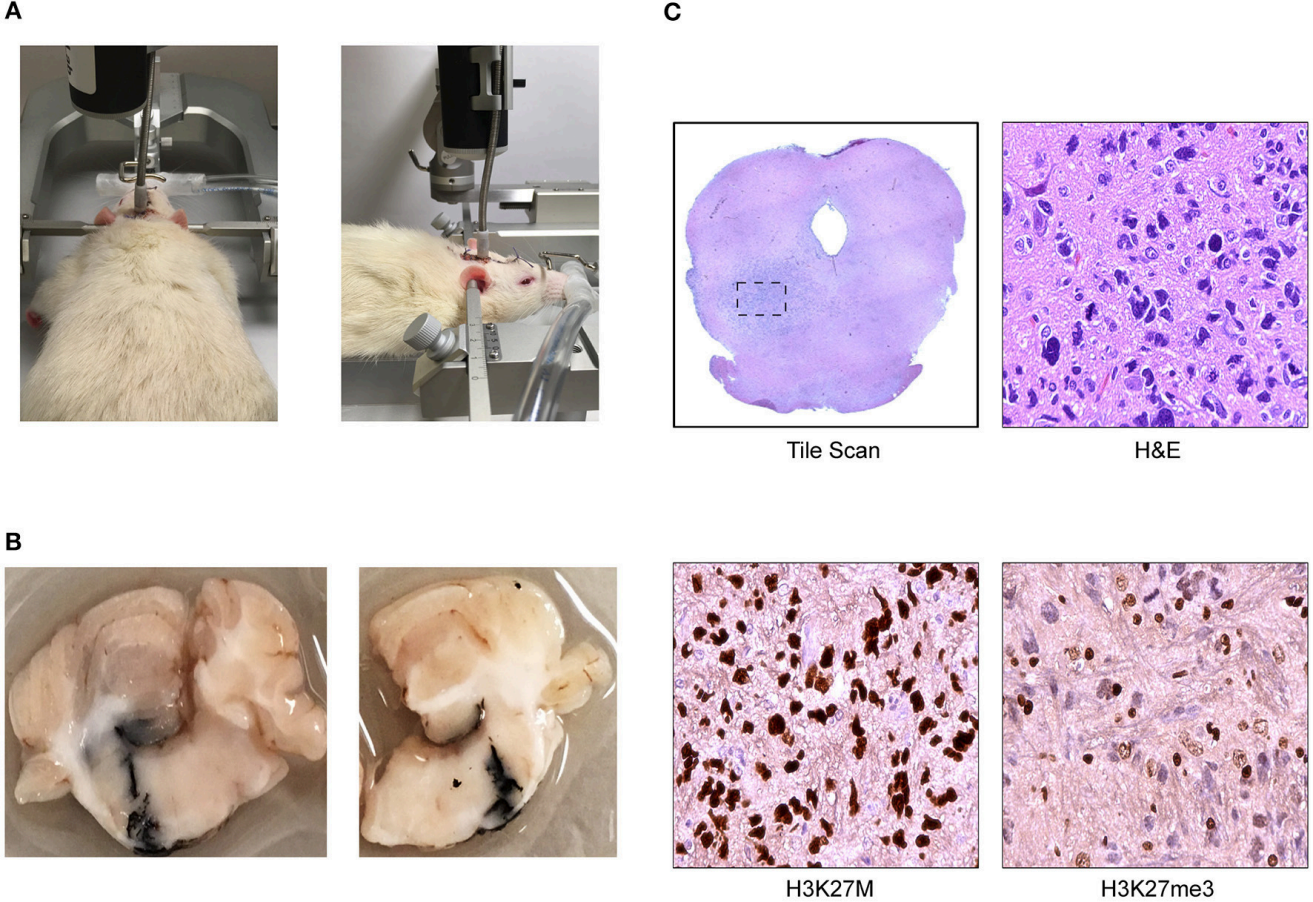
Cannula-guided convection enhanced delivery in the rat pons (Daniels Laboratory—Mayo Clinic). **(A)** Infusion pump is attached to the cannula installed on rat brain where the infusate was delivered at a constant rate over time. **(B)** Photograph of ink solution injected at 8 mm of depth with a Hamilton syringe through the cannula validating V_d_. **(C)** Coronal section of athymic nude rat brainstem with DIPG patient derived xenograft showing representative images of low magnification scan of H&E and high magnification scan of H3K27M and H3K27me3 immunohistochemical (IHC) staining.

In using CED to treat DIPG, effective placement of the infusing catheter is a critical step, given the need for the catheter to be fixed in a stable position over a prolonged period, the challenge in placing the catheter into the brainstem without creating a neurologic deficit, and positioning the catheter in such a way as to allow treatment of the entire tumor with infusate. Long-term catheter placement of CED in the brainstem of rodents and primates has been successfully carried out by a number of groups (Figure 2), reviewed extensively by Goodwin et al (49, 50). Large human trials involving CED for supratentorial high grade glial tumors have demonstrated an ability to place catheters for treatment in humans safely, though the most notable example, the PRECISE trial, did not monitor distribution of drug over the course of therapy (51). Further, in the PRECISE trial, catheter placement was scored based on depth from brain surface, distance from pial surfaces, and distance from resection cavity/ependymal surface, and only 51% of catheters had adequate placement (52). However, these criteria to determine adequate placement have not been prospectively validated (53). More recently, CED catheters have now been placed into the brainstem in humans and, recent studies have shown this technique to be safe (54–57). Much of the foundational work in this area has been conducted in Bristol, UK. Baura and colleagues used robotic assistance to place a catheter for CED carboplatin treatment in a large pontine tumor in a 5 year-old patient and were able to achieve infusate to 95% of the tumor (57). This group has also worked to develop bone-anchored ports and multiple-catheter systems (up to four catheters), allowing for chronic intermittent CED to a highly-tailored area (58, 59). Improved stereotactic placement of catheters and increasing use of stereotactic biopsy in obtaining tissue for diagnosis and study in DIPG placement has also increased facility and demonstrated the safety of these techniques, which require similar expertise and carry similar attendant risks as CED treatment to the brainstem (60, 61). To validate the real-world application of CED to the pons, Souweidane and colleagues report their results of the first Phase I trial in DIPG tumor patients. CED of the radionuclide [^124^I]-8H9 for treatment of DIPG in 28 patients was well-tolerated without any dose-limiting toxicities observed in the study, with one patient experiencing transient hemiparesis (trial NCT01502917) (54).

### Variables in CED

CED is a robust and tunable platform allowing for infusion of a range of agents of varying sizes over a range of tissue volumes. Such malleability requires optimization for a given therapeutic agent in order to achieve optimal delivery, however. The volume of tissue treated depends on the volume and rate of infusion, with most CED studies utilizing rates between 0.5 and 6 μL/min (56). These characteristics are essential for ensuring an adequate volume of distribution (V_d_) and avoiding reflux into the infusing catheter. The tissue being treated dictates these parameters to a large degree—tumors frequently have high interstitial pressures that need to be overcome in order to achieve adequate infusate delivery, and this resistance can drive infusate away from the desired region (53). Tumors also lack normal vasculature, further exacerbating this outward-directed pressure gradient that can drive the dispersion of drug delivered to the tumor bed (53, 62). The size and shape of the infusing catheter is critical for optimizing both the degree of tissue penetration and avoiding backflow. While many studies have been conducted with traditional end port cannulas, improved infusion profiles have been observed with porous tipped catheters and those with a step-off design, reviewed in detail by Lewis et al. (63). Lewis et al. have also recently described a recessed step catheter that allows for “controlled reflux” of infusate, and may allow for even more tailored delivery of therapeutics moving forward (64). The properties of the infusate itself and its therapeutic payload are also critical variables, and will be discussed in detail below (64).

### Advantages for Drug Delivery

In light of the myriad variables in developing an effective CED platform, developing a CED platform for use in real-world situations is an ongoing challenge. However, there are key advantages to this technique that make its use appealing. Most notably in treating patients, ensuring the safety and reliability of these systems is critical. While select cases have made use of CED to the brainstem, the unique properties of every infused therapeutic makes the volume of distribution hard to predict (53). However, in cases where CED infusion in the pediatric brainstem resulted in neurologic changes, cessation of the infusion halted these effects (53, 65). As discussed above, reliable catheter placement remains an area of ongoing growth, but the ability to administer prolonged indwelling infusions via CED has been well-established. Treating a large volume of tissue with a relatively small amount of infusate is advantageous, particularly in treating DIPG, where the volume of therapeutic that can be infused may be limited due to tumor location. Particularly for larger molecules, CED can result in a V_d_ many times what would be predicted by diffusion alone (47, 53). CED allows for a homogenous distribution of infusate as well, ensuring the targeted area receives therapeutic levels of the administered drug (63).

## Properties of Infusate

The CED cannula itself, the volume of infusate (V_i_), and the rate of infusion are not the only critical factors in effective CED administration—the drug and infusate itself must be optimized for ideal distribution. Most critically, the drug infused but be optimized for CED. Size is a critical factor; as smaller molecules will distribute more readily through tissue (47). Mechanisms of clearance include active transport by various ATP-binding cassette (ABC) transport proteins, or CSF spaces that rapidly clear infusate (53, 66, 67). Interstitial pressures in tumors may also be higher than in the surrounding tissue, generating an outward pressure gradient leading to increased clearance of infusate (25, 68). Nano-scale particles (< 100 nm) seem to be the ideal size for achieving a large V_d_ /V_i_ ratio (67). Hydrophobic molecules also struggle with achieving large V_d_ when administered via CED, as do those that are positively charged (67). Surface modification of drugs, such as coating relatively hydrophobic molecules with albumin, can improve V_d_ as well (67, 69). Development of liposomal or nanoparticle formulations of drugs in order to improve CED pharmacokinetic profiles is an active area of development, and some current clinical trials are underway utilizing such formulations (70). Such formulations allow for controlled release of therapeutic over time, prevent premature degradation of drug, and allow for hydrophobic medications to traverse the extracellular space (70). Coupling drugs that are inherently nonspecific for tumor cells, such as toxins, chemotherapeutics, or radionuclide, to tumor-specific antibodies is another promising strategy, adding a degree of tumor specificity (71). Lastly, the viscosity of the infusate itself can be adjusted for improved CED. In some cases, increasing the viscosity of the carrier fluid can improve the V_d_ of drug, and can readily be achieved by the addition of sucrose or polyethylene glycol (PEG) (67, 72, 73). This is likely due to more efficiently convective forces in higher viscosity fluids, as low viscosity fluids may be more likely taken up by surrounding cells or reflux into the catheter (46, 73).

## Visualization of CED

The ability to accurately track the distribution of drug administered via CED is an essential challenge in advancing the methodology to clinical applications. Some therapeutics, particularly radionuclides, maybe tracked by positron-emission tomography/CT (PET/CT) in order to evaluate the volume of tissue treated by the therapeutic being administered (71). However, most small molecule or nanocarrier-packaged therapeutics administered by CED lack such an intrinsic ability to be tracked on imaging. Older studies made use of infusion-associated T2 signal changes on MRI to evaluated the area of tissue treated (57). As reviewed in detail recently by Lonser, many current studies co-administer a gadolinium agent such as Gd-DTPA in the infusate with the therapeutic, allowing for visualization of the area treated by CED via MRI (74). Similarly, iodine-based contrast agents such as iopamidol and iopanoic acid can be used for CT-based imaging of CED (74). However, as has been discussed, substances of differing sizes, charge, and hydrophobicity can have very different V_d_ when administered with a given V_i_, and so the use of co-administered gadolinium may not accurately reflect the distribution of the therapeutic agent. Efforts have been made to administer surrogate agents of similar size to the therapeutic agents being administered—Szerlip and colleauges co-infused viral particles and the iron-based contrast agent ferumoxtran-10, both ~24 nm in size, for imaging via MRI (75). However, such an approach still makes use of a surrogate marker for visualization.

## Current Animal Models For CED

**Figure 3:**
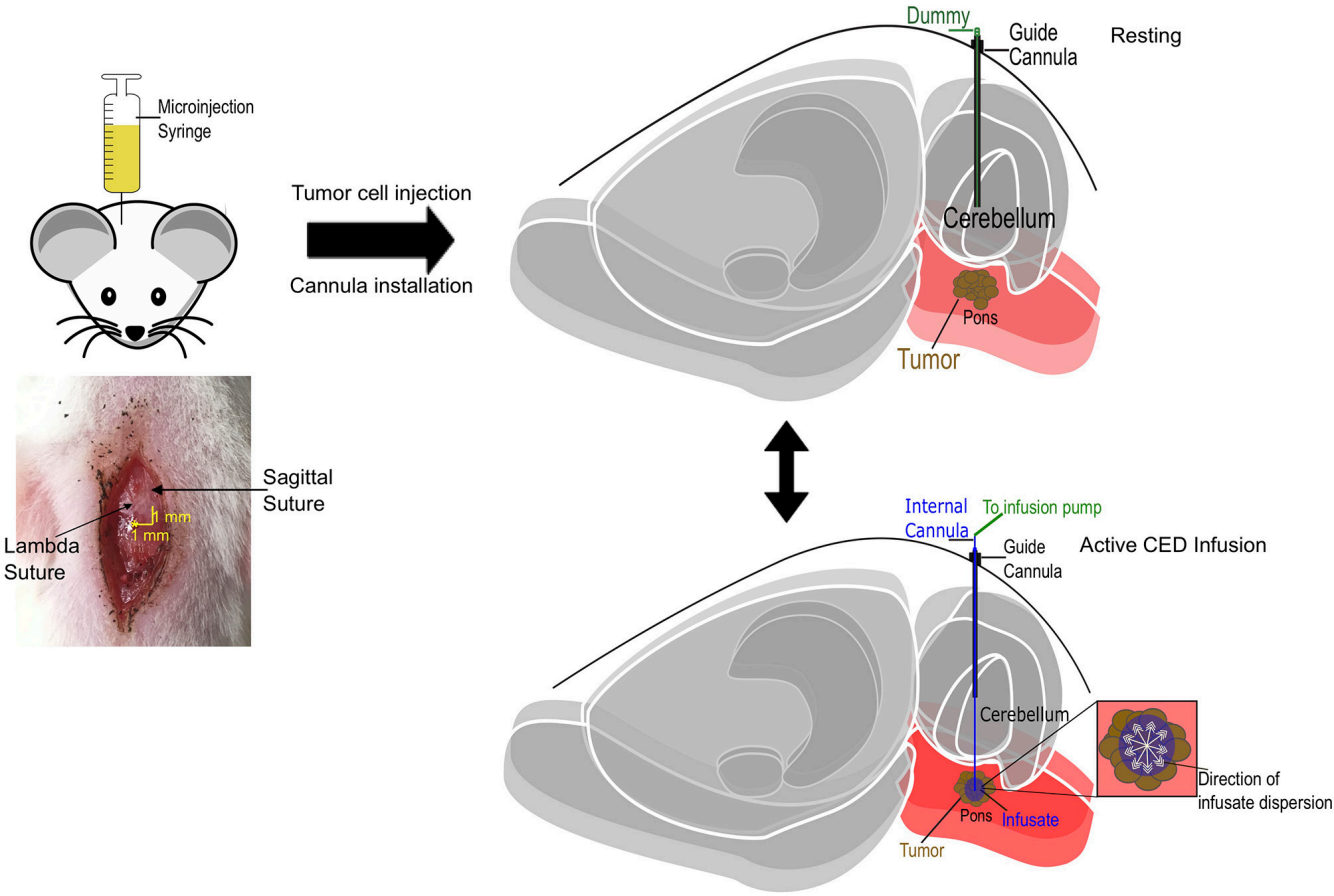
Schematic diagram of cannula-guided convection-enhanced delivery in rat. Cannula and tumor cell implantation coordinates in relation with lambdoid (1 mm lateral) and sagittal (1 mm posterior) sutures. The guide cannula is implanted into the animal post tumor cell implantation at 6 mm below the pedestal. A dummy cannula is inserted into the guide to protect the brain when there is no infusion (resting). During drug delivery, the dummy cannula is replaced with an internal cannula that projects 8 mm into the brain and the input end is connected with a microinjection syringe infusion pump that deliver infusate at a fixed rate.

A number of well-established models have been developed for studying CED. Rodent models in mice and rats (Figure 2) have been utilized for some time, as have models in larger organisms including pigs and primates (67, 76–79). A schematic model of CED in the mouse pons is diagrammed in Figure 3. Studying the dynamics of CED in these larger systems is critical in order to study distribution volumes at a scale relevant to human therapy. This is not only a function of size, but as discussed previously, CED bulk flow dynamics behave differently in different brain regions, particularly gray vs. white matter (46). Mice and rat brains have particularly limited amounts of white matter, limiting the generalizability of CED data derived from these models (67). A number of brainstem-specific models of CED have also been developed (49). Occhiogrosso et al. demonstrated that long term (24 h infusion) CED to the rodent brainstem was feasible (77). Sewing and colleagues demonstrated the ability to deliver carmustine via CED in the mouse brainstem (78). Zhou and colleagues have demonstrated the ability to infuse therapeutic agents, including kinase inhibitors, to the mouse brainstem with a favorable toxicity profile (80). Developing effective animal models of DIPG has also been an area of active development. Tumor models to study CED in animal models have also been developed, with much work done in the rat glioma models, including the F98 and 9L glioma lines (81, 82). However, more recent efforts have focused on developing brainstem-specific models to better study DIPG. Inoculating tumors in an anatomic position in these models is a challenge given the size and fragility of the brainstem, particularly in a small animal model, however several groups have successfully done so (49, 78). More recently, a genetically engineered mouse model of brainstem glioma has been developed driven by the H3K27M mutation, overexpression of platelet-derived growth factor (PDGF), and loss of p53 (83). Such a model, with *in situ* formation of tumors in the brainstem, may provide a critical tool for evaluating CED of therapeutics in a physiologically-relevant setting.

## Current Clinical Trials For DIPG Using CED

### ^124^I-8H9

Recent clinical trials in CED for brain tumors have been reviewed extensively by Zhou and colleges and Healey, therefore, a select few trials will be discussed here (46, 53). NCT01502917 is an ongoing phase I dose escalation study, open since 2011, evaluating CED delivery of ^124^I-8H9, a radionuclide-antibody complex directed against B7-H3, a surface marker expressed on the majority of DIPG tumors (46). This study applies a number of the key principles reviewed thus far, using CED of large molecules (antibodies in this case) to achieve a large volume of distribution, reporting a V_d_ /V_i_ ratio of 2.5 to 3.0. Dosimetry is effectively monitored with MRI imaging and V_d_ confirmed with the use of a radionuclide (46, 71). Thus far, the authors report no dose-limiting toxicities in 20 patients treated (46).

### Panobinostat

Panobinostat is a general histone deacetylase (HDAC) inhibitor that has shown good *in vitro* efficacy against DIPG tumors harboring the H3K27M mutation and, interestingly, those tumors without the mutations (22, 64). Orally-administered panobinostat for treatment of DIPG has been attempted, but the drug has known limitations in penetrating the BBB (64, 83, 84). A nanoparticle formulation of the drug, MTX110, as demonstrated a favorable toxicity profile when administered to the brainstem via CED in a rodent model (85). A human Phase I trial for CED of MTX110 opened in humans in May 2018 and is currently enrolling patients with newly diagnosed DIPG with or without biopsy (NCT03566199).

### Liposomal Irinotecan

Traditional chemotherapeutic agents are also being trialed for CED delivery to DIPG. Bruce and colleagues reported 2 cases of topotecan delivery via CED to the brainstem in two patients with DIPG (86). Patients underwent stereotactic biopsy of and placement of bilateral CED catheters, with one patient receiving drug treatment prior to radiation therapy and the other patient following completion of radiation. In both cases a modest reduction in tumor size was observed on MRI, and patients experienced worsening neurologic symptoms with high rates of infusion that improved with steroid used and cessation of infusion (86). In one case, infusion was resumed a lower rate following neurologic recovery and the patient tolerated this well (86). However, this study did not have an effective means to monitor the distribution of drug. Currently, a trial is enrolling using nanoliposomal irinotecan with gadolinium infusion for distribution monitoring (NCT03086616). This formulation allows for sustained release of drug over time and has shown some efficacy in rodent models when administered either via CED or intranasal (70).

### Multicatheter CED Injections

In an effort to achieve a more maximal and uniform V_d_ across heterogeneous tumors, Steven Gill et al. have developed a multiple CED catheter system placed with robotic assistance that connect to a single implanted manifold that can be infused intermittently (57). This system has the advantage of improved V_d_ due to multicatheter placement and the ability to chronically administer drugs of choice, however, the placement of 4 catheters in the brainstem increases the chances for neurological symptoms. They have published several preclinical studies in both small and large animal models, and are now utilizing this system in human patients (57–59). A four-port catheter system was used to treat a patient with recurrent glioblastoma with intermittent carboplatin infusions, with a subsequent reduction of tumor volume (59). The patient in this study ultimately succumbed to her disease 8 months following catheter implantation, but this case illustrates the feasibility of this approach in delivering a therapeutic payload.

### Future Directions

Future advancements in CED will come from multiple angles which include further refinements in hardware that have been discussed and an increase in our understanding of optimal drug characteristics for CED delivery which may include the development of CED specific chemotherapies. Robot assisted catheter placement for neurosurgical applications has already become common place for epilepsy procedures, and Renishaw has a robot system already on the market capable of delivering multi-brainstem CED catheters safely (87, 88). Probably more important than hardware technology is increasing our understanding of CED pharmacology. Most drugs that have been utilized for CED delivery have been selected based on anti-tumor efficacy in cell culture or animal models, without an understanding of CED pharmacology or convective kinetics. Studies that define optimal drug size, lipophilicity, status for brain efflux pumps and other important variables are required. In light of the myriad variables in delivering effective CED, developing a CED platform for use in real-world situations is an ongoing challenge and requires further studies. Next generation CED delivery for DIPG tumors will not only optimize the hardware for delivery, but the drugs being used.

## Conclusion

DIPG remains a devastating disease for which there is no effective treatment. This is due to the nature of the tumor itself and the anatomic location in which it occurs. There is now some promise in the development of targeted therapy, as the majority of these tumors harbor the H3K27M mutation; however, drug delivery remains a large hurdle. CED is an attractive means of delivering therapeutics to DIPG tumors, as it bypasses the BBB and allows for the treatment of a relatively large volume of tissue with small amount of infusate. This presents its own challenges as drug must be specifically formulated for optimal use via CED. There are several ongoing clinical trials investigating CED in DIPG treatment in humans and will hopefully offer hope to patients and families with this devastating disease.

## Author Contributions

DD provided direction. BH wrote the manuscript. LZ provided the figures. DD made revisions to the manuscript. All authors read and approved the final manuscript.

### Conflict of Interest Statement

The authors declare that the research was conducted in the absence of any commercial or financial relationships that could be construed as a potential conflict of interest. The handling editor declared a shared affiliation, though no other collaboration, with the authors at time of review.

## Abbreviations

DIPG: Diffuse intrinsic pontine glioma
CED: Convection-enhanced delivery
BBB: blood brain barrier.
